# Aspirin Eugenol Ester Protects Vascular Endothelium From Oxidative Injury by the Apoptosis Signal Regulating Kinase-1 Pathway

**DOI:** 10.3389/fphar.2020.588755

**Published:** 2020-11-20

**Authors:** Mei-Zhou Huang, Zhen-Dong Zhang, Ya-Jun Yang, Xi-Wang Liu, Zhe Qin, Jian-Yong Li

**Affiliations:** ^1^ Key Laboratory of New Animal Drug Project of Gansu Province, Key Laboratory of Veterinary Pharmaceutical Development of Ministry of Agriculture and Rural Affairs, Lanzhou Institute of Husbandry and Pharmaceutical Sciences of CAAS, Lanzhou, China; ^2^ Academician (Expert) Workstation of Sichuan Province, The Affiliated Hospital of Southwest Medical University, Luzhou, China

**Keywords:** aspirin eugenol ester, oxidative stress, apoptosis signal-regulating kinase-1, cardiovascular disease, apoptosis

## Abstract

Aspirin eugenol ester (AEE) is a new potential pharmaceutical compound possessing anti-inflammatory, anti-cardiovascular disease, and antioxidative stress activity. The pharmacological activities of AEE are partly dependent on its regulation of cell apoptosis. However, it is still unclear how AEE inhibits cell apoptosis on the basis of its antioxidative stress effect. This study aimed to reveal the vascular antioxidative mechanism of AEE in response to H_2_O_2_-induced oxidative stress in HUVECs and paraquat-induced oxidative stress in rats. In the different intervention groups of HUVECs and rats, the expression of ASK1, ERK1/2, SAPK/JNK, and p38 and the phosphorylation levels of ERK1/2, SAPK/JNK, and p38 were measured. The effects of ASK1 and ERK1/2 on the anti-apoptotic activity of AEE in the oxidative stress model were probed using the corresponding inhibitors ASK1 and ERK1/2. The results showed that in the HUVECs, 200 μM H_2_O_2_ treatment significantly increased the phosphorylation of SAPK/JNK and the level of ASK1 but decreased the phosphorylation of ERK1/2, while in the HUVECs pretreated with AEE, the H_2_O_2_-induced changes were significantly ameliorated. The findings were observed *in vitro* and *in vivo*. Moreover, inhibition of ASK1 and ERK1/2 showed that ASK1 plays a vital role in the protective effect of AEE on H_2_O_2_-induced apoptosis. All findings suggested that AEE protects the vascular endothelium from oxidative injury by mediating the ASK1 pathway.

## Introduction

Aspirin eugenol ester (AEE) is a newly discovered potential pharmaceutical compound possessing anti-inflammatory, anti-cardiovascular disease, and antioxidative stress pharmacological activities (Li et al., 2011; Li et al., 2012b; Ma et al., 2015; Ma et al., 2017; Huang et al., 2019a). In the H_2_O_2_-induced oxidative stress model using human umbilical vein endothelial cells (HUVECs), the protective effect of AEE on vascular oxidative injury is synchronized with the AEE-enhanced expression of Bcl2 and Nrf2 (Huang et al., 2019a; 2019c). It has been well documented that AEE can alleviate H_2_O_2_-induced dysfunction in mitochondria, the generation of ROS production and the increase in apoptosis rate by enhancing the expression of Bcl2 and Nrf2 (Huang et al., 2019a; 2019c). Mitochondrial dysfunction can induce the release of cytochrome C, apoptosis-inducing factor (AIF), and other factors into the cytoplasm to mediate downstream apoptotic signals that cause cell apoptosis (Chong et al., 2003; Chua et al., 2009; Choudhury et al., 2010; Cuadrado and Nebreda, 2010; Abate et al., 2019; Hasnat et al., 2019), and the exacerbation of reactive oxygen species (ROS) production induced by mitochondrial dysfunction is vital to cell apoptosis (Jaeschke and Bajt, 2006; Kim et al., 2006; Lai et al., 2012; Meng et al., 2018; Moradzadeh et al., 2018). In the signaling pathways of cell apoptosis, many signaling proteins, including those in the mitogen-activated protein kinase (MAPK) family, are vulnerable to ROS attack (Cuadrado and Nebreda, 2010; Son et al., 2011; Jung et al., 2014; Skala et al., 2016; Huang and Li, 2019). Whether MAPK signaling pathways are involved in the protective effect of AEE on H_2_O_2_-induced huvec apoptosis is unclear.

Apoptosis signal-regulating kinase 1 (ASK1), a member of the MAPK family, is a vital transmitter in the apoptosis signaling network (Ichijo et al., 1997; Tobiume et al., 2001). ASK1 is involved in various vascular disease signaling pathways (Papaconstantinou, 2019). In cerebral ischemia, ASK1 plays a key regulatory role in vascular permeability and edema formation (Song et al., 2015). The activity of ASK1 is subtly regulated primarily through the manipulation of scaffold adaptor proteins, such as thioredoxin (Trx) and 14-three to three protein, which are sensitive to ROS (Goldman et al., 2004; Fujino et al., 2007; Petrvalska et al., 2016). If excessive ROS accumulate in cells, ASK1 is inevitably inhibited, causing cell apoptosis (Fujino et al., 2007). Since AEE can reduce the H_2_O_2_-induced generation of ROS (Huang et al., 2019c), it is of interest to probe the effects of AEE on the signal pathways of ASK1 in an H_2_O_2_-induced apoptosis model.

Another member of the MAPK family, extracellular signal-related kinase (ERK), also plays a key role in the regulation of cell growth, development, division, and apoptosis. The activity of ERK1/2 is tightly regulated by upstream mitogen-activated protein kinase kinase (MAPKK) under physiological conditions, while the activity of ERK is affected by various pathological factors, such as viruses, bacteria and excessive ROS production, causing cell apoptosis (Sharma-Walia et al., 2005; Chen et al., 2019). The sensitivity of ERK1/2 to ROS is mainly accounted for by its cysteine residues (Romero et al., 2018; Huang and Li, 2019; Behring et al., 2020). Extensive research has demonstrated that oxidative stress can disrupt the activity of ERK1/2, causing cell apoptosis, and the anti-apoptotic activities of many antioxidant molecules are consistent with their effect on the activity of ERK1/2 (Jung et al., 2014; Shahin et al., 2018; Zhang et al., 2018; Lv et al., 2020). Previous studies have suggested that AEE possesses good antioxidant and anti-apoptosis activities; however, whether the ERK signaling pathway is involved in the antioxidant and anti-apoptosis processes of AEE remains unclear.

In this study, the effects of AEE on MAPK signaling pathways, including ASK1 and ERK signaling pathways, were investigated in an H_2_O_2_-induced oxidative stress model consisting of HUVECs and a paraquat (PQ)-induced oxidative stress model consisting of rats. It has been reported that PQ-induced toxicity in organisms is associated with the generation of free radicals and oxidative stress, causing severe injury to vascular, liver, lung and other tissues (Zhao et al., 2017; Eftekhari et al., 2018; Pang et al., 2019). The PQ-induced rat oxidative stress model has been widely used in research on PQ poisoning and antioxidant drugs (Eftekhari et al., 2018). The anti-apoptosis mechanism of AEE was explored on the basis of MAPK signaling pathways. This study investigates the molecular mechanism of AEE against vascular endothelial injury induced by oxidative stress and promotes the development of AEE as a potential drug for the treatment of vascular diseases.

## Materials and Methods

### Chemicals

Transparent crystals of AEE with a purity of 99.5% were prepared by RE-HPLC at the Lanzhou Institute of Husbandry and Pharmaceutical Sciences of CAAS. CMC-Na (0.5%) was used to suspend the AEE. An H_2_O_2_ solution (3%) and dimethyl sulfoxide (DMSO) were supplied by Sigma (St. Louis, MO). Trypsin-EDTA (0.05%), DMEM/F12 (1:1), and fetal bovine serum were obtained from Gibco (Grand Island, NY, USA). Hoechst 333342 staining solution, dihydroethidium (DHE) probe, a reactive oxygen species (ROS) assay kits, and a malondialdehyde (MDA) assay kit were purchased from Beyotime (Shanghai, China). MitoTracker Red CMXRos probe was purchased from Thermo Scientific (Waltham, MA, USA). Anti-ASK1, anti-ERK1/2, anti-SAPK/JNK, anti-SOD2, anti-p38, and superoxide dismutase (SOD) assay kits were obtained from Abcam (Cambridge, MA, USA), and anti-phospho-ERK1/2 (Thr202/Tyr204), anti-phospho-ASK1 (Thr838), anti-phospho-SAPK/JNK (Thr183/Tyr185), and anti-phospho-p38 (Thr180/Tyr182) antibodies were purchased from Cell Signaling Technology, Inc. (Beverly, MA, USA). An Annexin V/FITC apoptosis detection kit was acquired from BD Biosciences (San Diego, CA, USA). Lipofectamine 3,000 was obtained from Invitrogen (Waltham, MA, USA). shRNAs targeting ASK1 and ERK1/2 and control shRNA were obtained from GeneChem (Shanghai, China). An apoptosis signal-regulating kinase 1 (ASK1) enzyme-linked immunoassay kit and thioredoxin-1 (Trx1) enzyme-linked immunoassay kit were obtained from Shanghai Jianglai Industrial Limited By Share Ltd. (Shanghai, China). Selonsertib, AG-126, and ERK inhibitor (a thiazolidinedione compound) were purchased from APExBIO Technology LLC (Houston, Texas, USA). Methyl viologen dichloride (paraquat, PQ) was purchased from Aladdin (Shanghai, China). Human umbilical vein endothelial cells (HUVECs) (ATCC CRL-4053™) were purchased from ATCC (Rockville, MD, USA). Xanthine oxidase, lactic acid and sVCAM-1 enzyme-linked immunoassay kits were obtained from Shanghai Enzyme-linked Biotechnology Co., Ltd. (Shanghai, China). The LDH, CK, AST, ALT, BUN and Cr kits were acquired from Ningbo Meikang Biotechnology Co., Ltd (Ningbo, China).

### Cell Culture and Treatments

The protocol for cell culture and treatments was based on descriptions in our previous studies (Huang et al., 2019a; 2019c). In brief, HUVECs were cultured with DMEM/F12 (1:1) containing 10% fetal bovine serum, subcultured with 0.05% trypsin-EDTA, and then randomly divided into three groups: the normal group, the model group and the AEE pretreatment group. Cells in the normal group were incubated with culture medium. The model group was incubated with culture medium containing 200 µM H_2_O_2_ for 22 h. In the AEE pretreatment groups, cells were preincubated with culture medium containing different concentrations of AEE (0.5, 1, or 2 µM) for 24 h and then incubated with medium containing 200 µM H_2_O_2_ for 22 h.

### Animal Experiment

Thirty male specific pathogen-free (SPF) Sprague-Dawley (SD) rats (6 weeks old) weighing 120–130 g were purchased from the Laboratory Animal Center of Lanzhou Veterinary Research Institute (Lanzhou, China). All animals were housed in groups in SPF-class laboratory housing at a controlled relative humidity (55–65%), 12 h light/dark cycle and temperature (24 ± 2°C). Feed and drinking water were supplied to the SD rats ad libitum. The rats were randomly assigned to six groups (*n* = 6): 1) the control group, in which rats were administered equivalent saline by intraperitoneal injection (ip); 2) the PQ group, in which rats were administered PQ (20 mg/kg body weight, ip) and administered 0.5% CMC-Na (0.01 ml/g body weight) by gavage once a week for two weeks; 3) the AEE group, in which rats were preadministered AEE (27 mg/kg/day, 54 mg/kg/day, 108 mg/kg/and day body weight) by gavage once a day for two weeks before being administered PQ, and the AEE daily gavage treatment was stopped at the time of PQ administration. The body weight of each rat was measured daily and statistically analyzed once a week. All of the animals survived to the end of the study. The rats were then sacrificed by injecting pentobarbital (30 mg/kg body weight). Blood samples were collected from the heart into vacuum tubes. Serum samples were obtained by incubating the tubes at room temperature for 30 min and centrifuging at 4,000 rpm for 10 min at 4°C. The samples were then stored at −80°C until analysis. The aortas were carefully isolated from the rats for analysis of ROS and oxidative stress-related factors and protein measurement. All experimental protocols and procedures were approved by the Institutional Animal Care and Use Committee of Lanzhou Institute of Husbandry and Pharmaceutical Science of the Chinese Academy of Agricultural Sciences (approval no. NKMYD201907018; approval date: July 18, 2019). Animal welfare and experimental procedures were performed strictly in accordance with the Guidelines for the Care and Use of Laboratory Animals issued by the US National Institutes of Health.

### Measurement of Biochemical Profile in Rat Serum

LDH, CK, AST, ALT, BUN and Cr in serum were measured by an automatic biochemical detector with corresponding commercial kits.

### Measurement of Xanthine Oxidase, Lactic Acid and sVCAM-1 in Rat Serum

The levels of xanthine oxidase, lactic acid and sVCAM-1 in rat serum were measured by the corresponding commercial kits according to the manufacturer’s protocols. In brief, all reagents were maintained at room temperature before use. The serum samples or standard working solution samples were added to the corresponding micro ELISA plate wells and combined with the appropriate specific antibody. Then, a biotinylated detection antibody and avidin-horseradish peroxidase (HRP) conjugate were added successively to each microplate well and incubated. Free components were washed away. The substrate solution was added to each well and incubated. The enzyme-substrate reaction was terminated by the addition of stop solution. The optical density (OD) was measured spectrophotometrically at a wavelength of 450 nm.

### Measurement of Intracellular Reactive Oxygen Species and Mitochondrial Membrane Potential

A reactive oxygen species assay kit and MitoTracker Red CMXRos probe were used to measure intracellular ROS and mitochondrial membrane potential. In brief, HUVECs were cultured in a 24-well glass bottom cell culture plate and treated following the description in *Cell Culture and Treatments*. Then, the cells were incubated with phenol-free red medium containing MitoTracker Red CMXRos probe (10 μM) and DCFH-DA probe (5 nM) at 37°C for 25 min in the dark. The relative fluorescence intensity of the cells was measured using a laser scanning confocal microscope (ZEISS LSM-800, Jena, Germany).

### Measurement of Reactive Oxygen Species in the Aorta

The level of ROS in the rat aortas was detected by a dihydroethidium (DHE) probe according to the manufacturer’s protocols. In brief, fresh aortas were weighed, ground using a glass grinder, and the tissue was centrifuged at 4,000 rpm for 10 min at 4°C. The supernatant coupled with DHE working solution was added to the 96-well plate and incubated at 37°C for 20 min, and the fluorescence density was measured by an Enspire microplate reader at an excitation wavelength of 560 nm and an emission wavelength of 610 nm.

### Measurement of Apoptosis Signal-Regulating Kinase 1 Levels in Cells and Rat Serum

The level of ASK1 in cells and rat serum was measured an ASK1 ELISA kit according to the manufacturer’s protocols. The serum samples or standard working solution samples with the specific antibody for ASK1 were added to the micro ELISA plate wells. Then, a biotinylated detection antibody and avidin-horseradish peroxidase (HRP) conjugate were added successively to each microplate well and incubated. Free components were washed away. The substrate solution was added to each well and incubated. The enzyme-substrate reaction was terminated by the addition of stop solution. The optical density (OD) was measured spectrophotometrically at a wavelength of 450 nm.

### Measurement of the Superoxide Dismutase, Thioredoxin-1 and Malondialdehyde Levels in Rat Serum

The levels of MDA, Trx1 and SOD in rat serum were assessed using the corresponding commercial kits according to the manufacturer’s protocols. The serially diluted MDA standards and test serum samples were prepared, and 10 μL of MDA color reagent stock solution was added to each well of the MDA standard and serum sample and incubated at room temperature for 20 min. Ten microliters of reaction solution was added to each plate cell and incubated at room temperature for 60 min. The absorbance increase in each well was monitored by an Enspire microplate reader at 695 nm. The protocols for detecting Trx1 and SOD were similar to those of ASK1, as explained in *Measurement of Intracellular ROS and Mitochondrial Membrane Potential*.

### Protein Expression Analysis

The expression of ASK1, ERK1/2, SAPK/JNK, p38, phospho-ERK1/2, phospho-SAPK/JNK, and phospho-p38 among different treatments for HUVECs was assessed by Western blot analysis. In brief, the total protein of the cells was extracted using RIPA, quantified by the bicinchoninic acid (BCA) method, and separated on a precast SDS-PAGE gel (4–20%). The separated proteins were transferred onto polyvinylidene fluoride (PVDF) membranes using standard procedures. The protein blots were incubated with the corresponding primary antibodies and internal control *β*-actin and then incubated with HRP-conjugated secondary antibody. The results were detected using a BIO-RAD ChemiDoc™ MP imaging system (Alfred Nobel Drive Hercules, California, USA) and normalized to the corresponding internal control *β*-actin to eliminate variations in total protein.

The expression of ASK1 in the HUVECs after different treatments s was evaluated by cell immunofluorescence analysis. Briefly, the cells were cultured on glass coverslips, fixed with 4% paraformaldehyde, permeabilized using 0.1% Triton-100, and labeled with a primary antibody against ASK1. Then, the cells were incubated with an Alexa Fluor^®^ 488-conjugated secondary antibody. The relative fluorescence intensity of the cells was measured by laser scanning confocal microscopy (ZEISS LSM-800).

The expression of SOD2, ASK1, phospho-ERK1/2, phospho-SAPK/JNK, and phospho-p38 in aortas was evaluated by Western blot analysis. In brief, the total protein in the aortas was extracted using a total protein extraction kit, quantified by the bicinchoninic acid (BCA) method, and separated on a precast SDS-PAGE gel (4–20%). The separated proteins were transferred onto polyvinylidene fluoride (PVDF) membranes using standard procedures. The protein blots were incubated with the corresponding primary antibodies and internal control *β*-actin and then incubated with HRP-conjugated secondary antibody. The results were detected using a Bio-Rad ChemiDoc™ MP imaging system (Alfred Nobel Drive Hercules, California, USA) and were normalized to the corresponding internal control β-actin to eliminate variations in total protein.

### Intervention of the Apoptosis Signal-Regulating Kinase 1 and ERK1/2 Signaling Pathways

The ASK1 and ERK1/2 signaling pathways of HUVECs were disrupted by inhibitors corresponding to ASK1 and ERK1/2. In brief, HUVECs were cultured with DMEM/F12 (1:1) containing 10% fetal bovine serum, subcultured with 0.05% trypsin-EDTA, and then randomly divided into six groups: normal group, model group, intervention group of ASK1-expressing cells, intervention group of ERK1/2-expressing cells, intervention group of ASK1-expressing cells treated with AEE, and intervention group of ERK1/2-expressing cells treated with AEE. The cells in the normal group were incubated with culture medium. In the model groups, the cells were incubated with culture medium containing 200 µM H_2_O_2_ for 22 h. In the intervention group, the ASK1-expressing cells were preincubated with culture medium containing an ASK1 inhibitor and for 40 min and then incubated with medium containing 200 µM H_2_O_2_ for 22 h. In the intervention group, the ERK1/2-expressing cells were preincubated with culture medium containing an ERK1/2 inhibitor for 40 min and then incubated with medium containing 200 µM H_2_O_2_ for 22 h. In the intervention group, the ASK1-expressing cells with AEE treatment were preincubated with culture medium containing AEE for 24 h, and then, the culture medium was replaced with medium containing an ASK1 inhibitor and incubated for 40 min, and finally, the cells were incubated with medium containing 200 µM H_2_O_2_ for 22 h. In the intervention group, ERK1/2-expressing cells with 1.0 µM AEE treatment were preincubated with culture medium containing AEE for 24 h, and the medium was replaced with culture medium containing an ASK1 inhibitor and incubated for 40 min, and finally, the cells were incubated with medium containing 200 µM H_2_O_2_ for 22 h.

In addition, the ASK1 and ERK1/2 signaling pathways in the HUVECs were inhibited by short hairpin RNA (shRNA) against ASK1 and ERK1/2. According to the manufacturer'|’s instructions, shRNAs targeting ASK1 and ERK1/2 or control shRNA (GeneChem, China) were transfected using Lipofectamine 3,000 (Invitrogen, USA) to downregulate ASK1 and ERK1/2 in the HUVECs, and then, these HUVECs were treated with 1.0 μM AEE and H_2_O_2_ according to the protocol described in *Cell Culture and Treatments*. The silencing efficiency of the short hairpin RNAs (shRNA) against ASK1 and ERK1/2 was confirmed by Western blotting (Supplementary Figure S1).

The changes in the apoptosis rate were detected in the different intervention groups by an Annexin V/PE apoptosis detection kit and flow cytometry. In brief, HUVECs were cultured in a 6-well glass-bottom cell culture plates and treated following the description in *Cell Culture and Treatments*. Then, the cells were collected, washed three times with cold PBS, stained with PE-Annexin and 7-ADD for 20 min at room temperature in the dark, and analyzed using flow cytometry. The following controls were used to establish compensation and quadrants: unstained cells, cells stained with PE-Annexin V and cells stained with 7-ADD.

### Statistical Analysis

All experiments and data analysis were performed according to blinding principles. Statistical analysis was carried out using SAS 9.2 (SAS Institute Inc., NC, USA). All data are presented as the means ± SD. The differences between intervention groups were analyzed with one-way ANOVA followed by Duncan’s multiple comparisons test. Statistical significance was defined as *p* < 0.05. The statistical analyses were applied to selected pairs.

## Results

### Aspirin Eugenol Ester Ameliorated the Changes in Body Weight, Biochemical Profile and Disease-Related Factors in Paraquat-Induced Rats

PQ can cause dysfunction of calcium pumps and mitochondria, inhibit lactic acid oxidation and gluconeogenesis, and reduce lactic acid clearance, which results in the promotion of xanthine oxidase (XO) production and the accumulation of lactic acid (LA). To explore the effect of AEE on the PQ-induced toxic effects of organisms, tissues and cells, the levels of xanthine oxidase, lactic acid and sVCAM-1 in rat serum were detected. As shown in Table 1, after treating the rats with PQ, the body weight of the rats decreased significantly. Pretreatment of the rats with AEE (54 mg/kg body weight) for 2 weeks significantly ameliorated the decrease in body weight induced by PQ. In addition, as shown in Table 2, after treating the rats with PQ, the levels of xanthine oxidase (XO), lactic acid (LA) and sVCAM-1 in rat serum increased significantly. Pretreatment of the rats with AEE significantly ameliorated the aforementioned PQ-induced changes in the rats. These results suggest that AEE can significantly ameliorate the PQ-induced toxic effects in organisms. Moreover, to explore the effect of AEE on the PQ-induced toxic effects, physiological biochemical parameters were measured. The results showed that the physiological biochemical parameters, including lactic dehydrogenase (LDH), creatine kinase (CK), aspartate transaminase (AST), alanine aminotransferase (ALT), blood urea nitrogen (BUN) and Cr (creatinine), were significantly changed, and the dysregulation of these biochemical parameters was significantly ameliorated in the AEE pretreatment group (the data are provided in Supplementary Table S1). The findings suggested that AEE can relieve the PQ-induced toxic effects in the heart, liver and kidney.

**TABLE 1:** Body weight (BW) changes of the rats in different intervention groups (Mean ± SD).

Group	BW prior to administration of PQ	BW after administration of PQ	Changes in BW
Control	311.3 ± 20.0	322.3 ± 14.8	16.0 ± 1.7
PQ	323.3 ± 15.6	284.1 ± 15.9	−39.2 ± 5.7*
27 mg/kg AEE + PQ	313.8 ± 8.9	294.9 ± 9.6	−18.8 ± 1.4^#^
54 mg/kg AEE + PQ	316.4 ± 10.4	320.4 ± 6.5	−2.7 ± 5.2^#^
108 mg/kg AEE + PQ	326.0 ± 8.9	327.3 ± 10.26	1.3 ± 1.6^#^

* p < 0.05 compared with the normal group.

# p < 0.05 compared with the PQ group.

**TABLE 2:** The levels of xanthine oxidase, lactic acid and sVCAM-1 in rat serum (Mean ± SD).

Group	XO (Ng/ml)	LA (Mmol/L)	sVCAM-1 (ng/ml)
Control	76.4 ± 2.2	0.79 ± 0.05	48.3 ± 4.2
PQ	193.5 ± 32.6*	4.66 ± 0.30*	194.7 ± 25.6*
27 mg/kg AEE + PQ	114.4 ± 17.4^#^	1.07 ± 0.23^#^	55.3 ± 8.2^#^
54 mg/kg AEE + PQ	52.6 ± 6.1^#^	0.52 ± 0.09^#^	45.5 ± 7.9^#^
108 mg/kg AEE + PQ	53.9 ± 5.2^#^	0.37 ± 0.03^#^	170.3 ± 13.7^#^

* p < 0.05 compared with the normal group.

# p < 0.05 compared with the PQ group.

### Aspirin Eugenol Ester Mitigated the Dysfunction of Reactive Oxygen Species and the Mitochondrial Membrane Potential Induced by H_2_O_2_

**FIGURE 1:**
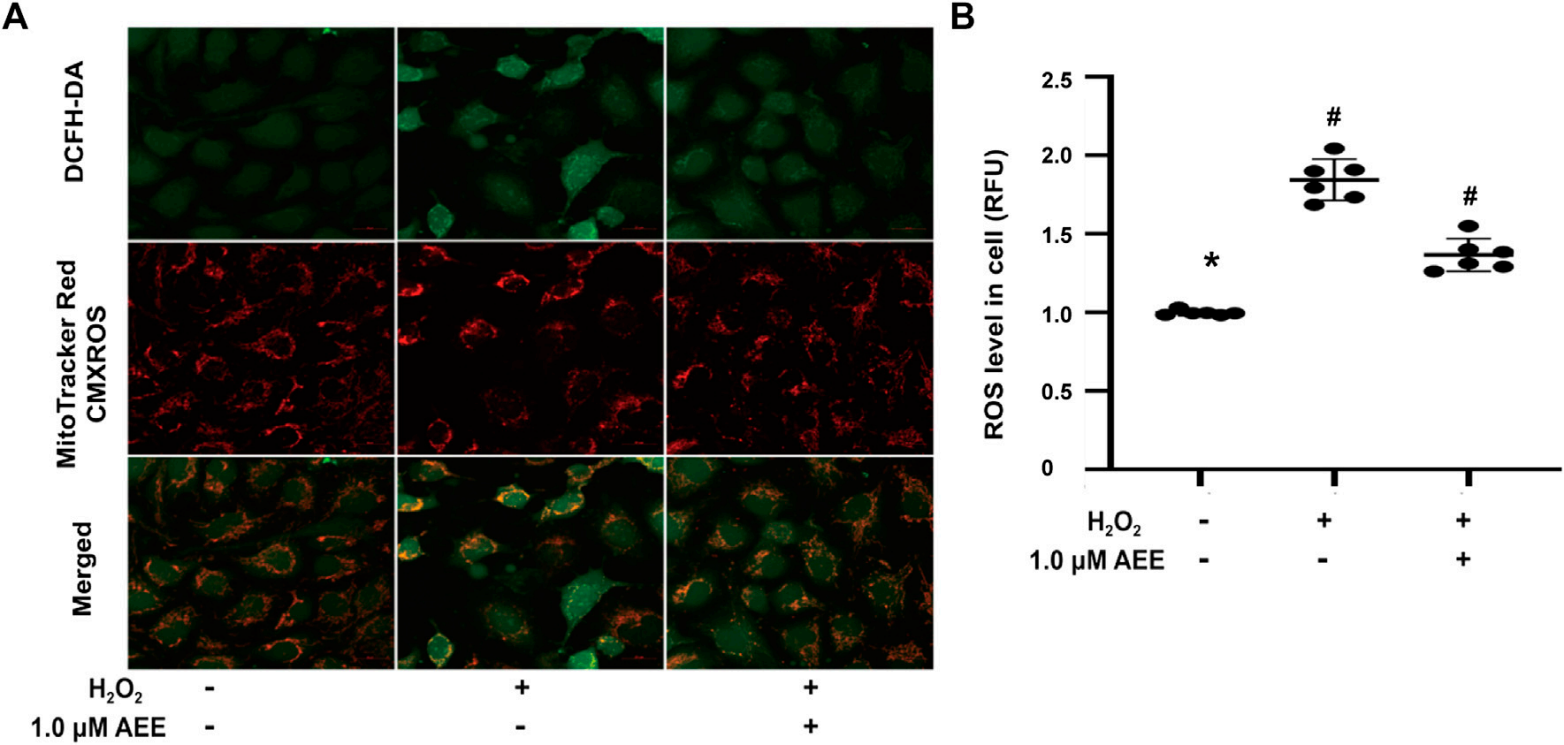
AEE ameliorated the dysfunction of mitochondria, manifested as the collapse of the membrane potential and increase of reactive oxygen species induced by H_2_O_2_; values are presented as the means ± SD where applicable (*n* = 6). All data were normalized to the corresponding control and reported in relative units (RU). *p < 0.05 compared with the normal group, ^#^ *p* < 0.05 compared with the H_2_O_2_ group. “+”: with the treatments in the HUVECs; “−”: without the treatments in the HUVECs.

To verify the changes in the redox status of the HUVECs in different treatment groups, the cellular ROS and mitochondrial membrane potential were measured. As shown in Figure 1, after exposure to 200 μM H_2_O_2_ for 22 h, the mitochondrial membrane potential of HUVECs decreased significantly, and cellular ROS levels increased significantly. Pretreatment of the HUVECs with 1.0 μM AEE for 24 h significantly ameliorated the decrease in mitochondrial membrane potential and the increase in cellular ROS induced by H_2_O_2_.

### Aspirin Eugenol Ester Strengthened the Antioxidant Ability in Paraquat-Induced Rats

**FIGURE 2:**
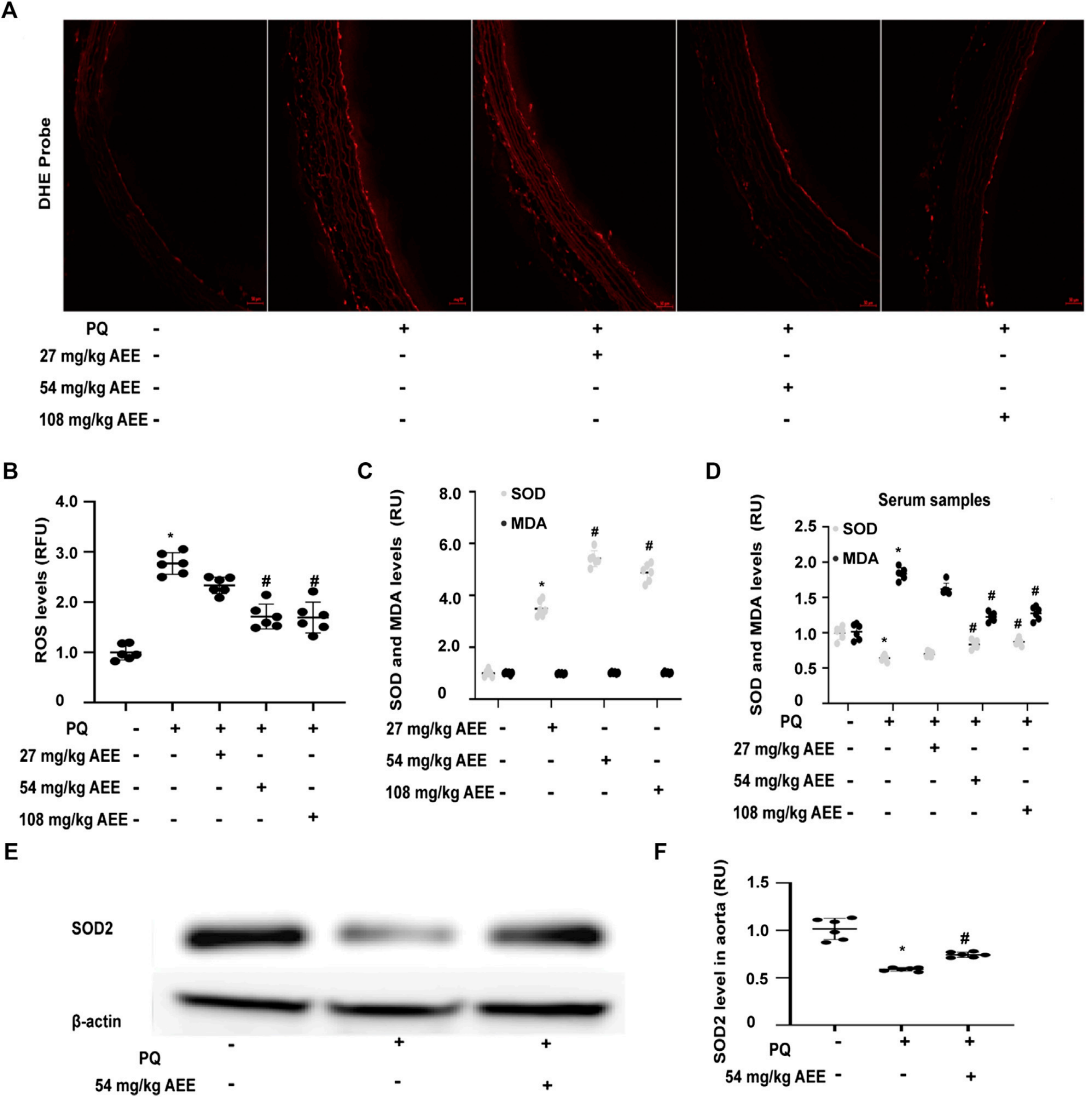
AEE reduced the PQ-induced increase in ROS in the aorta by enhancing the antioxidant ability of the rat. **(A, B)** AEE reduced the PQ-induced increase in ROS in the aorta; values are presented as the means ± SD where applicable (*n* = 6). **(C, D)** AEE ameliorated the levels of SOD and MDA in serum without PQ treatment or with PQ treatment; values are presented as the means ± SD where applicable (*n* = 6). **(E, F)** AEE reduced the PQ-induced decrease in SOD2 in the aorta. All data were normalized to the corresponding control and reported in relative units (RU). *p < 0.05 compared with the normal group, ^#^ *p* < 0.05 compared with the PQ group. “+”: with the treatments in the rats; “−”: without the treatments in the rats.

To determine whether AEE can also affect the level of ROS in the aorta of the PQ-induced rats, ROS were measured in the presence and absence of AEE. As shown in Figures 2A,B, the level of ROS in the aorta of PQ-induced rats was higher than that in the control rats (*p* < 0.05). Pretreatment of the rats with AEE (54 mg/kg body weight) for 2 weeks significantly ameliorated the increase in ROS induced by PQ in the aorta.

SOD, a key antioxidant enzyme, plays a vital role in the regulation of the oxidation steady state. Oxidative stress caused by a plethora of pro-oxidant factors is closely related to the regulation of SOD. Moreover, SOD is also a pivotal participant in the transduction of oxidative stress signaling. MDA is a hallmark of oxidative stress and is used to assess the oxidation status of the body and the antioxidant capacity of drugs. To detect the effect of AEE on the antioxidant ability, the SOD and MDA levels in the AEE-pretreated rats without and with PQ treatment were evaluated. As shown in Figures 2C,D, there was no difference in the levels of MDA between the control rats and AEE-treated rats in the absence of PQ. Treatment of the rats with PQ significantly enhanced the levels of MDA. However, the stimulatory effect of PQ on MDA was significantly reduced by pretreating the rats with 54 mg/kg/day AEE for 14 days. After the rats were pretreated with AEE for 14 days in the absence of PQ, the activity of SOD was significantly increased. Pretreating the rats with AEE for 14 days significantly mitigated the activity of SOD induced by PQ. Moreover, treatment of rats with PQ significantly decreased the expression of SOD2 in the aorta. After the rats were pretreated with AEE for 14 days, the PQ-reduced expression of SOD2 in the aorta was significantly ameliorated (Figures 2E,F). These findings suggest that AEE can reduce the stimulatory effect of PQ on rats by strengthening the antioxidant ability.

### Aspirin Eugenol Ester Ameliorated the Stimulatory Effect of H_2_O_2_ and Paraquat on Mitogen-Activated Protein Kinase Family-Related Proteins

**FIGURE 3:**
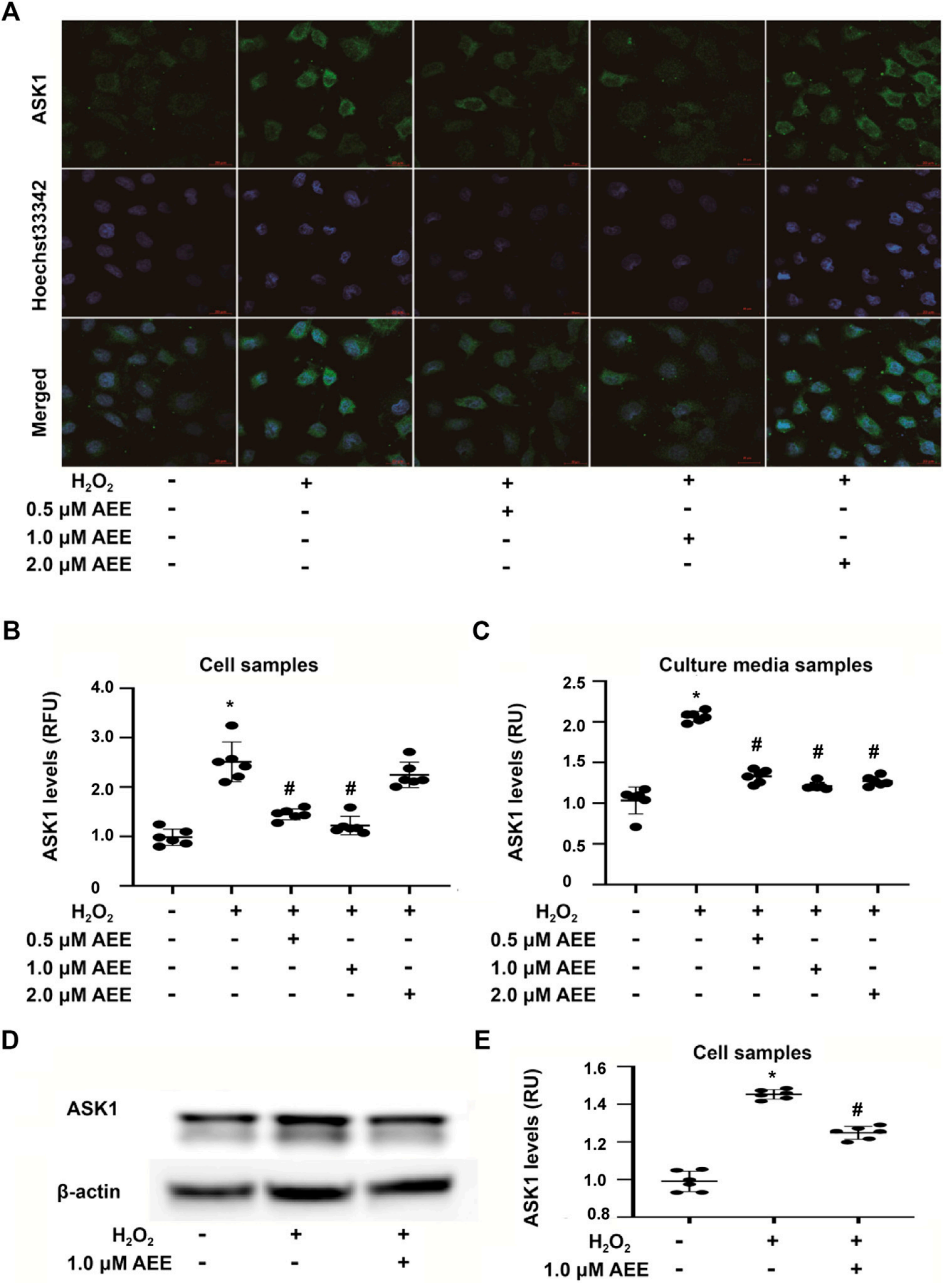
AEE attenuated H_2_O_2_-induced expression of ASK1 in the HUVECs. **(A, B)** AEE reduced H_2_O_2_-induced expression of ASK1 in the HUVECs; values are presented as the means ± SD where applicable (*n* = 6). **(C)** AEE weakened the increase in ASK1 induced by H_2_O_2_ in culture media **(D, E)** AEE reduced H_2_O_2_-induced expression of ASK1 in the HUVECs. All data were normalized to the corresponding control and reported in relative units (RU). *p < 0.05 compared with the normal group, ^#^ *p* < 0.05 compared with the H_2_O_2_ group. “+”: with the treatments in the HUVECs; “−”: without the treatments in the HUVECs.

**FIGURE 4:**
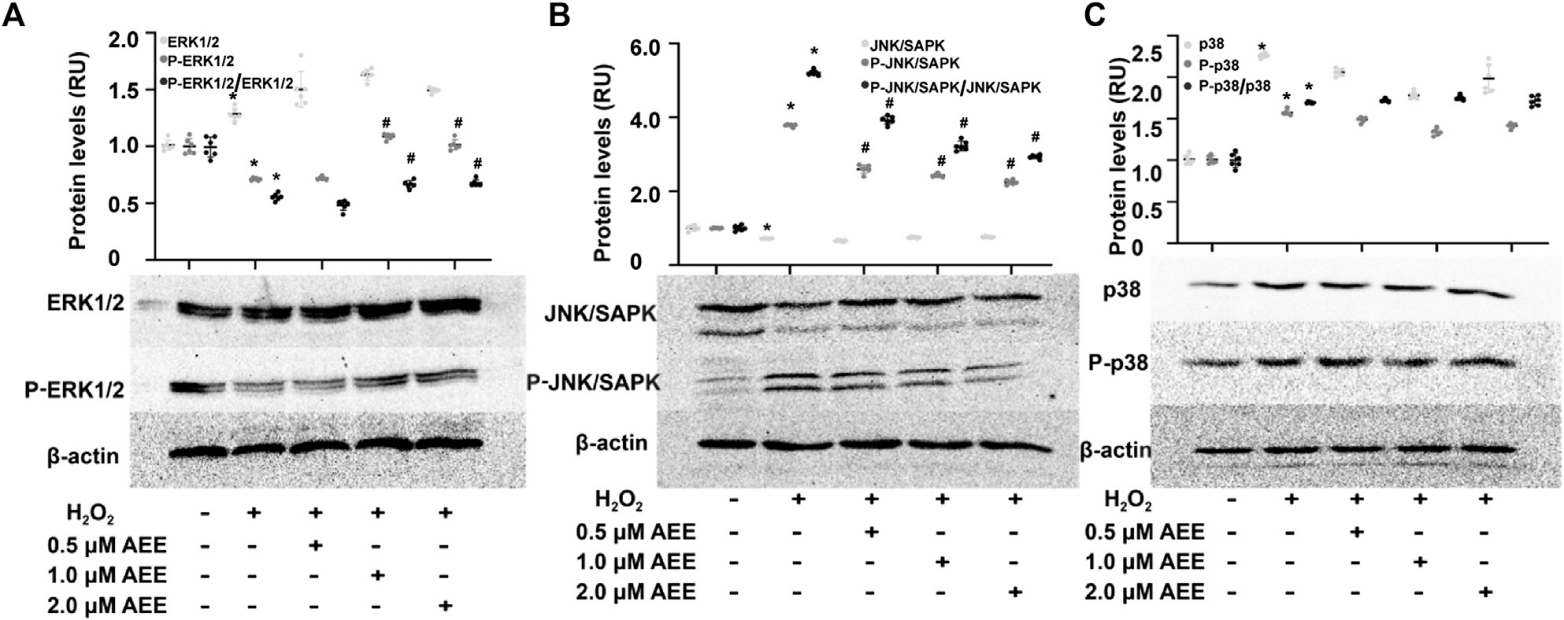
AEE ameliorated H_2_O_2_-induced changes in apoptosis-related proteins. **(A–C)** AEE reduced the H_2_O_2_-induced expression of ERK1/2, P-ERK1/2, SAPK/JNK, P-SAPK/JNK, p38 and P-p38 in the HUVECs. Values are presented as the means ± SD where applicable (*n* = 6); all data were normalized to the corresponding control and reported in relative units (RU). *p < 0.05 compared with the normal group, ^#^ *p* < 0.05 compared with the H_2_O_2_ group. “+”: with the treatments in the HUVECs; “−”: without the treatments in the HUVECs.

To determine the effect of AEE on MAPK family-related proteins in *in vivo* and *in vitro* models, changes in ASK1, ERK1/2, SAPK/JNK, p38, phospho-ERK1/2, phospho-SAPK/JNK, and phospho-p38 levels were detected by Western blotting, immunofluorescence assays and ELISAs. Changes in JNK, p38 and ERK following exposure to H_2_O_2_ were assessed at multiple time points (0.5, 2, 8 and 16 h). The results showed that the activation of JNK and ERK did not change in the 2 h following exposure to H_2_O_2_ and that after exposure to H_2_O_2_ for more than 8 h, there were significant changes in the phosphorylation levels of JNK, p38 and ERK in the HUVECs in different treatment groups. In addition, the phosphorylation level of p38 was increased after treating the HUVECs with H_2_O_2_ for 2 h. These data are provided in the Supplementary Figure S2. As shown in Figures 3A,C, 200 μM H_2_O_2_ treatment significantly increased the expression of ASK1 in the HUVECs and the level of ASK1 in the cell culture supernatant, while pretreating the HUVECs with 1.0 μM AEE significantly ameliorated the H_2_O_2_-enhanced expression of ASK1. Hoechst signals were also attenuated, and the ASK1 signals were suppressed, as shown in Figure 3A. To confirm, the ASK1 level data acquired by the cell immunofluorescence protocol for the different treatment groups were reliable, the expression of ASK1 was measured by Western blotting. As shown in Figures 3D,E, the results of ASK1 expression as detected by Western blotting were consistent with the results detected by immunofluorescence with the HUVECs. In different treatment groups, differential cellular status might affect Hoechst staining, but the exact mechanism leading to Hoechst signal changes is still unclear. The stimulating effect of oxidation factors on vascular endothelial cells results in an increase in cell membrane permeability and leakage of cytoplasmic protein. In particular, *in vitro*, cytoplasmic proteins are released from the cytoplasm of apoptotic cells into the culture medium. ASK1, a cytoplasmic protein, might be released into the extracellular space when vascular endothelial cells are injured. Therefore, the level of ASK1 in the culture medium and serum was measured. We speculated that extracellular ASK1 levels following H_2_O_2_ stimulation might reflect secretion from dead/dying cells. Furthermore, in the HUVECs, 200 μM H_2_O_2_ treatment significantly increased the phosphorylation levels of SAPK/JNK and p38 but decreased the phosphorylation levels of ERK1/2, while pretreatment of the HUVECs with 1.0 μM AEE significantly ameliorated H_2_O_2_-induced changes in ERK1/2 and SAPK/JNK (Figure 4). Phospho-ASK1 (Thr838) was detected to measure the activity of ASK1. The results showed that pretreating the HUVECs with 1.0 μM AEE significantly attenuated the H_2_O_2_-enhanced phosphorylation level of ASK1 (the data are provided in the Supplementary Material).

**FIGURE 5:**
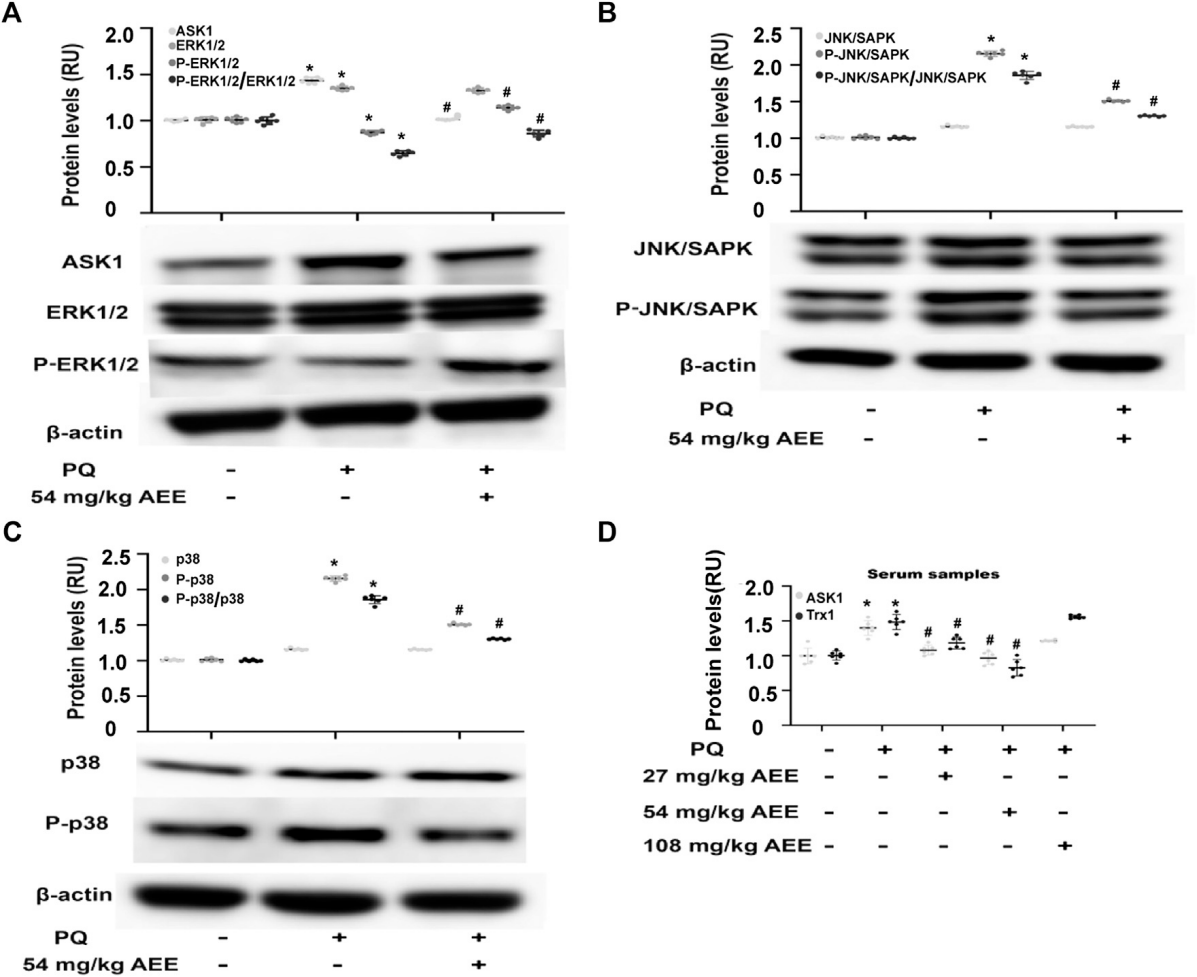
AEE ameliorated PQ-induced changes in apoptosis-related proteins in the aorta. **(A–C)** AEE reduced the PQ-induced expression of ASK1, p38, P-p38, SAPK/JNK, P-SAPK/JNK, ERK1/2 and P-ERK1/2 in the aorta. Values are presented as the means ± SD where applicable (*n* = 6). **(D)** AEE reduced the increase in ASK1 and Trx1 in serum induced by PQ. Values are presented as the means ± SD where applicable (*n* = 6); all data were normalized to the corresponding control and reported in relative units (RU). *p < 0.05 compared with the normal group, ^#^ *p* < 0.05 compared with the PQ group. “+”: with the treatments in the rats; “−”: without the treatments in the rats.

In the rats, PQ intervention significantly increased the phosphorylation level of SAPK/JNK and p38 in the aortas and the level of ASK1 in the serum and aortas and decreased the phosphorylation level of ERK1/2, while pretreatment of the rats with AEE for 2 weeks significantly ameliorated the aforementioned PQ-induced changes (Figures 5A–D). Trx-1, a redox-regulating protein with antioxidant activity, is induced by oxidative stress (Nakamura, 2004), and it is also an important adaptor of ASK1. Moreover, it has been reported that serum Trx-1 levels are recognized as an oxidative stress marker (Abdiu et al., 2000; Nakamura, 2004; Ohashi et al., 2006). As shown in Figure 5D, the changes in Trx1 in the serum were consistent with those of ASK1 following treatment of the rats with PQ and AEE. All the findings suggested that AEE can ameliorate the stimulatory effect of H_2_O_2_ and PQ on MAPK family-related proteins. In addition, there was no close dose dependence on the AEE effects in the regulation of MAPK family-related protein expression. AEE (1.0 μM) showed the optimal regulatory effect on H_2_O_2_-induced HUVECs, and pretreatment of the rats with AEE (54 mg/kg/day body weight) showed the optimal regulatory effect on PQ-induced rats.

### Apoptosis Signal-Regulating Kinase 1 Reduced the Effect of Aspirin Eugenol Ester on H_2_O_2_-Induced Apoptosis

**FIGURE 6:**
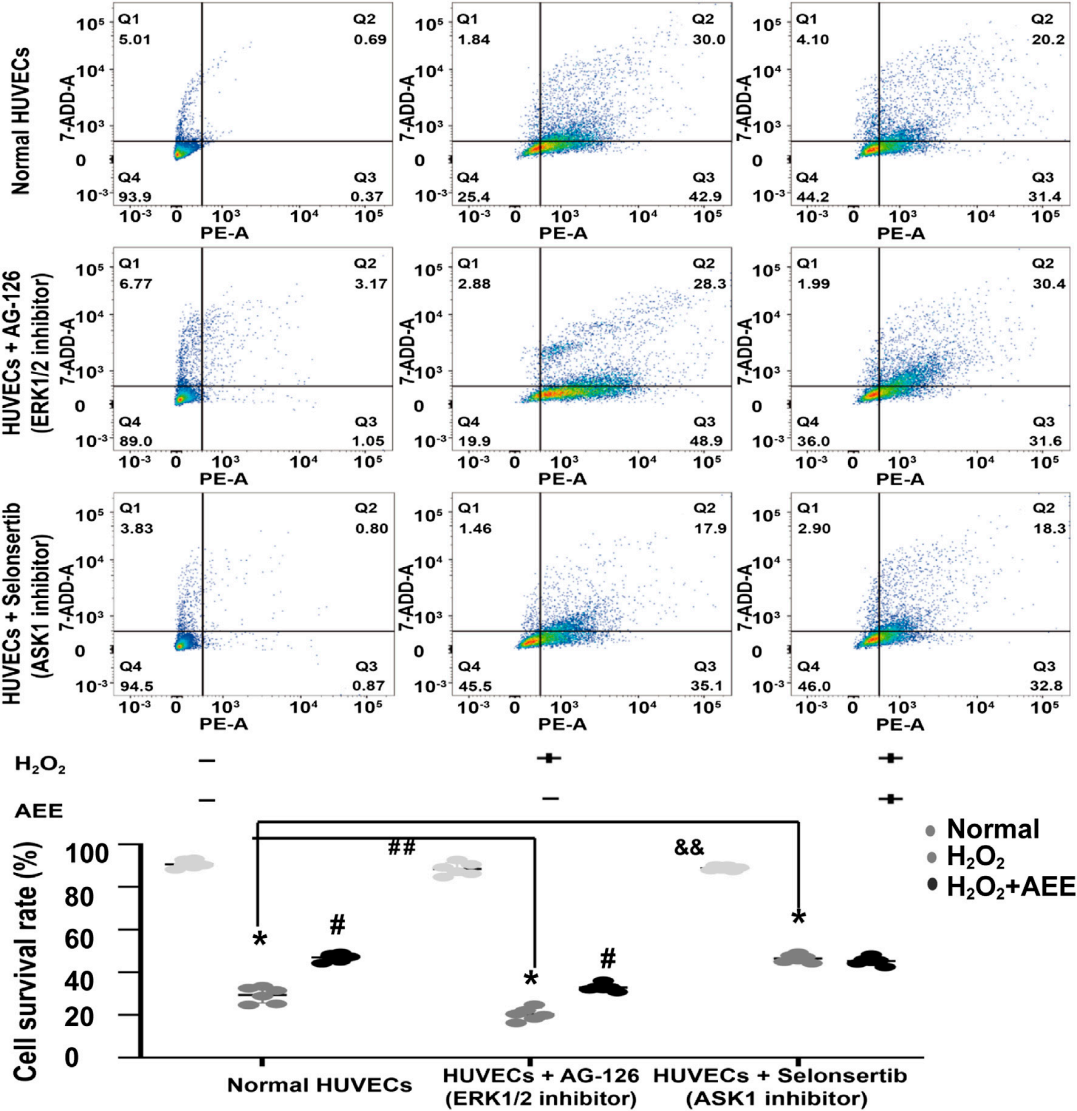
Intervention of ASK1 with an inhibitor reduced the effect of AEE on H_2_O_2_-induced apoptosis. Values are presented as the means ± SD where applicable (*n* = 6). *p < 0.05 compared with the normal group; ^#^ *p* < 0.05 compared with the H_2_O_2_ group; and *p* < 0.05. “+”: with the treatments in the HUVECs; “−”: without the treatments in the HUVECs.

**FIGURE 7:**
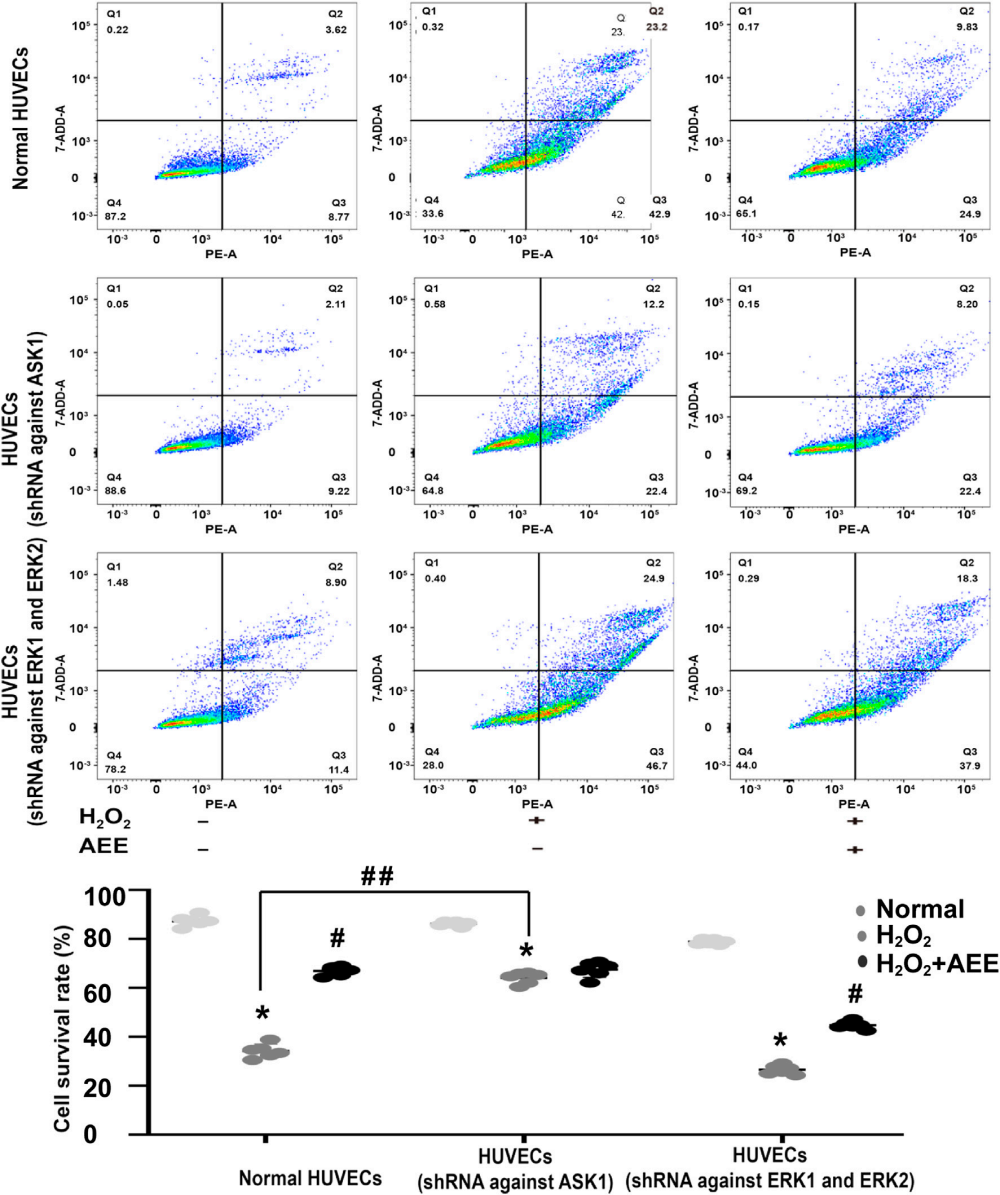
Intervention of ASK1 by shRNA reduced the effect of AEE on H_2_O_2_-induced apoptosis. Values are presented as the means ± SD where applicable (*n* = 6). *p < 0.05 compared with the normal group; ^#^ *p* < 0.05 compared with the H_2_O_2_ group; and *p* < 0.05. “+”: with the treatments in the HUVECs; “−”: without the treatments in the HUVECs.

To verify the role of ASK1 and ERK in the protective effect of AEE on H_2_O_2_-induced apoptosis, the corresponding inhibitors of ASK1 and ERK and shRNAs against ASK1 and ERK1/2 were used to inhibit the activity of ASK1 and ERK in the HUVECs, respectively. Cell apoptosis was approximately 75% after the HUVECs were exposed to 200 μM H_2_O_2_ for 22 h, whereas the cell apoptosis rate was reduced to 50% by AEE treatment. After treatment with the inhibitor of ASK1 and shRNA against ASK1, the cell apoptosis rate induced by H_2_O_2_ was significantly decreased, whereas there were opposite consequences when the HUVECs were treated with an inhibitor of ERK1/2 and shRNAs against ERK1/2, respectively. After the HUVECs with inhibited ERK1/2 were treated with 200 μM H_2_O_2_ for 22 h, the apoptosis rate significantly increased compared to that of normal HUVECs induced by H_2_O_2_. Pretreating ERK1/2-inhibited HUVECs with AEE significantly decreased the apoptosis rate compared to that of the ERK1/2-inhibited HUVECs induced by H_2_O_2_ (Figures 6, 7).

As shown in Figure 6, after the HUVECs with inhibited ASK1 were treated with 200 μM H_2_O_2_ for 22 h, the apoptosis rate significantly decreased compared to that of the normal HUVECs induced by H_2_O_2_. Pretreating ASK1-inhibited HUVECs with AEE did not significantly attenuate the apoptosis rate in the ASK1-inhibited HUVECs induced by H_2_O_2_.

## Discussion

AEE is a newly discovered potential pharmaceutical compound possessing anti-inflammatory, anti-cardiovascular disease, and antioxidative stress pharmacological activities (Li et al., 2012a; Li et al., 2012b; Karam et al., 2015). Previous studies have suggested that AEE can reduce H_2_O_2_-induced mitochondrial dysfunction, manifested as increased ROS and collapsed mitochondrial transmembrane potential, by regulating Bcl2 and Nrf2 (Huang et al., 2019b; 2019c). Consistent with these previous studies, the present study showed that AEE can ameliorate the increase in ROS induced by H_2_O_2_. Moreover, the results also showed that AEE can enhance the antioxidant ability of rats by increasing the level of SOD in serum and reducing the level of LA and XO in serum and ROS production in the aortas induced by PQ. The present study, in agreement with previous studies, confirmed that AEE can reduce ROS generation in an oxidative stress model.

A body of evidence has suggested that excessive ROS trigger the apoptotic signaling pathway centered in mitochondria (Papa and Skulachev, 1997; Lee et al., 2018; Zou et al., 2019). Furthermore, mitochondria are sensitive to ROS, and many other proteins and factors participating in the apoptotic signaling pathway are also sensitive to ROS, such as MAPK family proteins. ASK1 and ERK1/2, as members of the MAPK family, play vital roles in the apoptotic signaling pathway (Kadowaki et al., 2005; Hsieh and Papaconstantinou, 2006; Huang et al., 2018; Xu et al., 2018). In previous studies by our group, AEE attenuated the collapsed mitochondrial transmembrane potential induced by H_2_O_2_. ASK1, a potent ROS sensor, can sense the cellular redox status and channels cells with imbalanced oxidation status into apoptotic pathways (Huang and Li, 2019). In this study, ERK1/2, JNK or p38 were not activated in the first 2 h following H_2_O_2_ stimulation of HUVECs, which was a different result than that of previous studies. Several studies have reported that short-term (0.5 h or 4 h) H_2_O_2_ exposure can significantly activate apoptosis pathways of HUVECs, including the ASK1, ERK1/2 and JNK/SAPK signaling pathways (Wen et al., 2013; Jin et al., 2015; Patel et al., 2016; Wang et al., 2019). However, it has been well documented that the stimulation effect of H_2_O_2_ on HUVECs is dependent on dose and exposure time, and primed HUVECs are more sensitive to H_2_O_2_ than nonprime HUVECs (Patel et al., 2016). The dose of H_2_O_2_ ranging from 10 μM to 1 mM and the times for H_2_O_2_ exposure were found in published studies (Jin et al., 2015; Patel et al., 2016; Wang et al., 2019). We proposed that the origin of the HUVECs and the dose of H_2_O_2_ were the main reasons for the aforementioned substantial differences from published studies. In the present study, after the HUVECs were treated with 200 μM H_2_O_2_ for 22 h, the expression of ASK1 significantly increased compared to that in the normal HUVECs. Under physiological conditions, ASK1 is ubiquitinated and degraded once its physiological function is achieved (Matsuzawa et al., 2002; Sakauchi et al., 2017). However, when ASK1 is continuously activated, it is not immediately degraded, causing the accumulation of ASK1 in cells (Nagai et al., 2009; Abdel-Magid, 2019). Pretreating the HUVECs with AEE significantly decreased the expression of ASK1 compared to that of the HUVECs in the model group. It was proposed that in H_2_O_2_-induced HUVECs, the decrease in ASK1 expression by pretreatment with AEE is closely related to the weakening of the stimulatory effect of ROS induced by H_2_O_2_. In the *in vivo* experiment, AEE also ameliorated the increase in ROS, the levels of ASK1 and Trx1 in serum, and ASK1 expression in aortas induced by PQ. These findings were consistent with the results of *in vitro* experiments, which further supported our hypothesis that the effect of AEE on ASK1 was related to ROS. It was intriguing that in this study, the level of Trx1 in serum was increased, and the trend was consistent with that of ASK1 followed by treatment with PQ and AEE. In previous studies, it was reported that TNFα, hydrogen peroxide, UV, thermal shock and several other stimuli activated Trx transcription [37]. It is speculated that the increase in Trx in the PQ-induced rats was a consequence of oxidative stress compensation. While AEE at the 54 mg/kg/day dose generally conferred more protection than the 27 mg dose, the 108 mg dose was least effective on several parameters, such as sVCAM-1. This might indicate a narrow dose range for inducing the significant antioxidant stress effects *in vivo*.

To further verify the role of ASK1 in the effect of AEE on vascular oxidative injury, the downstream target molecules of ASK1 were detected. SAPK/JNK and p38, pivotal target molecules of ASK1, play key roles in the ASK1 pathway. It has been well documented that ASK1 could regulate the activity of SAPK/JNK and p38 by affecting their phosphorylation status (Nishitoh et al., 1998). In the present study, the phosphorylation of SAPK/JNK and p38 was increased in the HUVECs induced by H_2_O_2_ and in the aortas of the rats induced by PQ. However, pretreatment of the HUVECs or rats with AEE significantly attenuated the phosphorylation of SAPK/JNK induced by H_2_O_2_ or PQ. Based on these results and previous studies, it is speculated that in the H_2_O_2_-induced oxidative injury model of HUVECs and the PQ-induced oxidative stress model of rats, the increase in the phosphorylation of SAPK/JNK was due to ASK1 activation, and ASK1 played an important role in the protective effect of AEE on vascular oxidative injury. To confirm this speculation, the activity of ASK1 was inhibited in the HUVECs by a special inhibitor. Since pretreatment of the ASK1-inhibited HUVECs with AEE did not significantly attenuate the apoptosis rate in the ASK1-inhibited HUVECs induced by H_2_O_2_, it is suggested that ASK1 plays an indispensable role in the protective effect of AEE on vascular oxidative injury.

In addition, in the present study, the phosphorylation of ERK1/2 was lower in the HUVECs induced by H_2_O_2_ and in the aortas of the rats induced by PQ. However, pretreatment of the HUVECs or rats with AEE significantly reversed the changes induced by H_2_O_2_ or PQ. Many studies have reported that ERK1/2 are sensitive to ROS and play vital roles in the regulation of apoptosis and autophagy induced by oxidative stress. To further clarify the roles of ERK1/2 in the effect of AEE on vascular oxidative injury, the activity of ERK1/2 was inhibited by a special inhibitor in the HUVECs. Because pretreatment of ERK1/2-inhibited HUVECs with AEE significantly attenuated the apoptosis rate in the ERK1/2-inhibited HUVECs induced by H_2_O_2_, it is suggested that ERK1/2 might play a secondary role in the protective effect of AEE on vascular oxidative injury, which is different than the role of ASK1.

In the present study, all findings suggested that the MAPK pathway plays an important role in the protective effect of AEE on vascular oxidative injury. Previous studies showed that AEE relieved vascular oxidative injury by regulating NOS and the mitochondrial-lysosomal axis (Matsuzawa et al., 2002; Sakauchi et al., 2017). However, it is still unclear how these biological processes mutually regulate the protective effect of AEE on vascular oxidative injury. It is speculated that the effect of AEE on ROS and reactive nitrogen species (RNS) is a key method to regulate the aforementioned biological processes in vascular oxidative injury, but the subtle regulatory mechanism of the protective effect of AEE on vascular oxidative injury needs to be substantially explored. Moreover, after the analysis of all data from the present study, the relationship of the protective effect of AEE on PQ-induced oxidative stress with PQ elimination from the body remains unclear and is worthy of further study.

## Conclusion

Dysfunction of MAPK signaling pathways, including ASK1, is involved in vascular oxidative injury. AEE can effectively prevent vascular oxidative injury by reducing the accumulation of ASK1 and the phosphorylation of SAPK/JNK.

## Data Availability Statement

The original contributions presented in the study are included in the article/Supplementary Material, further inquiries can be directed to the corresponding author/s.

## Ethics Statement

The animal study was reviewed and approved by Institutional Animal Care and Use Committee of Lanzhou Institute of Husbandry and Pharmaceutical Science of Chinese Academy of Agricultural Sciences.

## Author Contributions

JYL and MZH designed and performed the experiments and wrote the manuscript. ZDZ performed partly the experiments. YJY synthesized and purified AEE. XWL and ZQ assisted with the animal experiments. JYL supervised the study and revised the manuscript.

## Conflict of Interest

The authors declare that the research was conducted in the absence of any commercial or financial relationships that could be construed as a potential conflict of interest.
