# Supplementary material for: Aspirin Eugenol Ester Protects Vascular Endothelium From Oxidative Injury by the Apoptosis Signal Regulating Kinase-1 Pathway
Source: Front Pharmacol. 2020 Nov 20;11:588755. doi: 10.3389/fphar.2020.588755 (PMC7919194; doi:10.3389/fphar.2020.588755)
Supplement: Supplementary file 1 [file DataSheet1_v1.PDF]

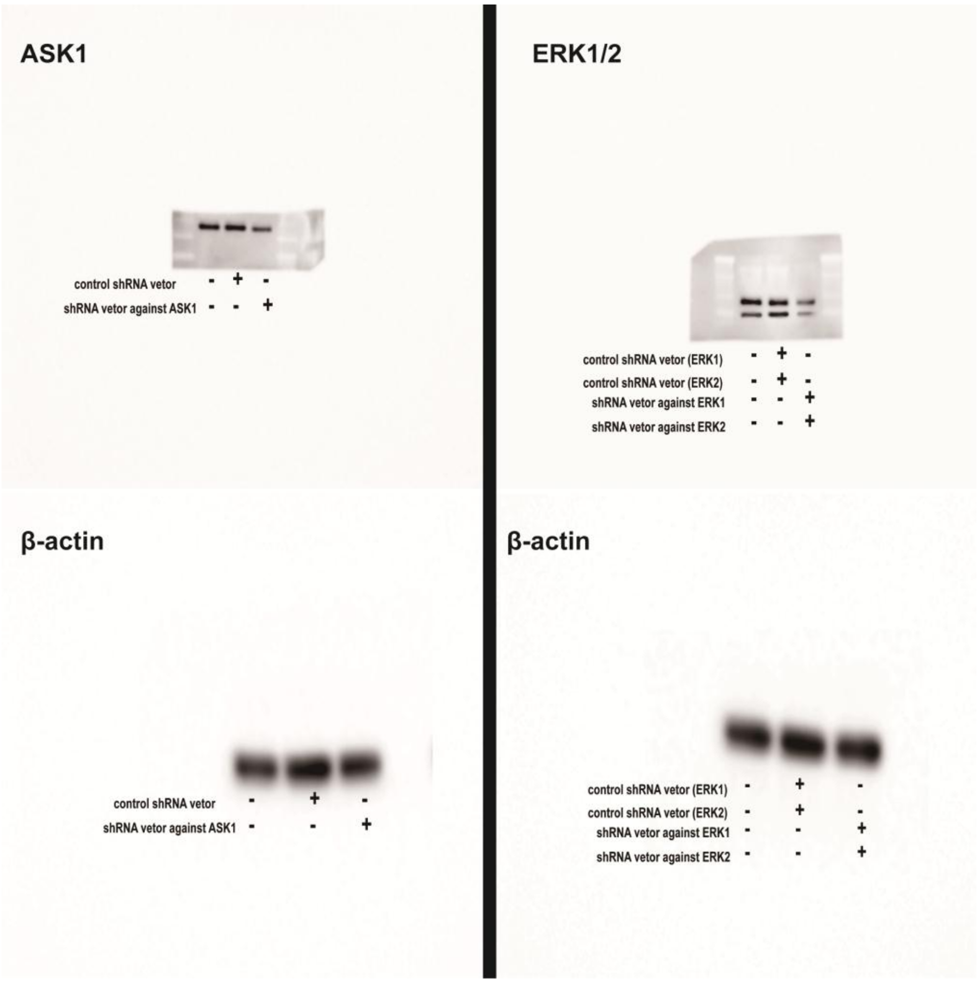

Figure S1 Transfection of HUVEC with corresponding shRNA to inhibit ASK1 and ERK1/2.

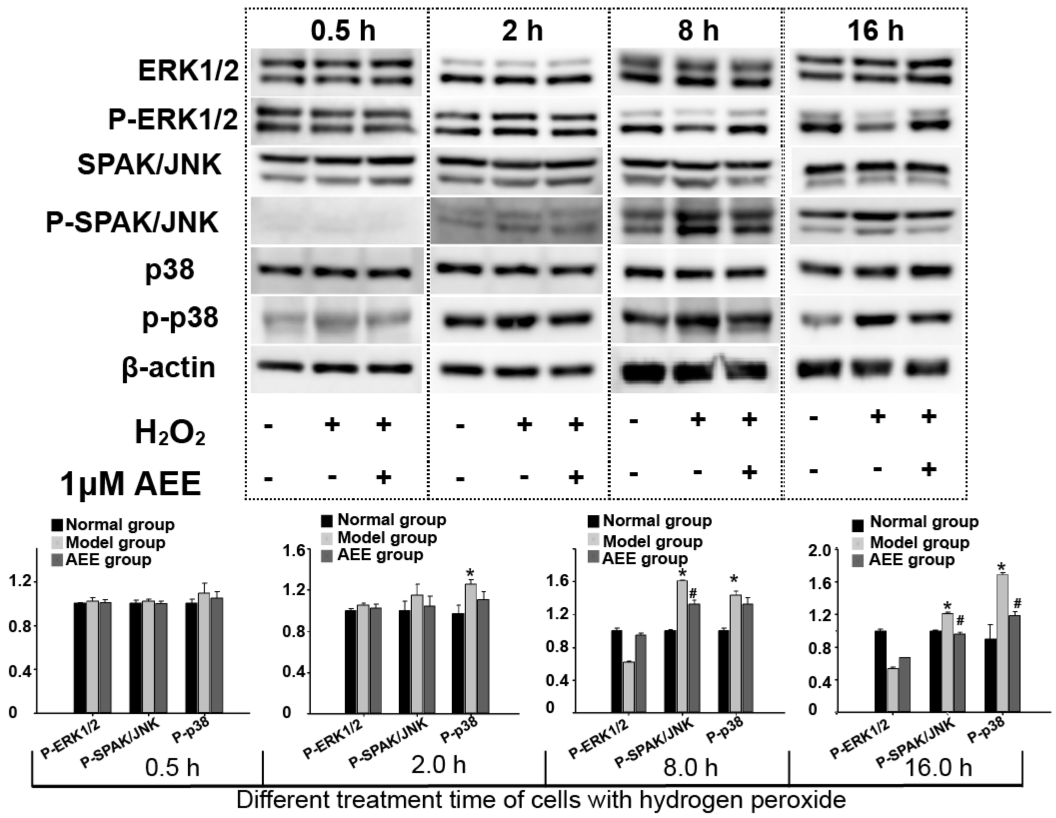

Figure S2 The changes in the activation of JNK, p38 and ERK following exposure to H_2O_2 for different time.*$p < 0.05$ compared with the normal group; #$p < 0.05$ compared with the H_2O_2 group. "+": with the treatments in the HUVECs; "–": without the treatments in the HUVECs.

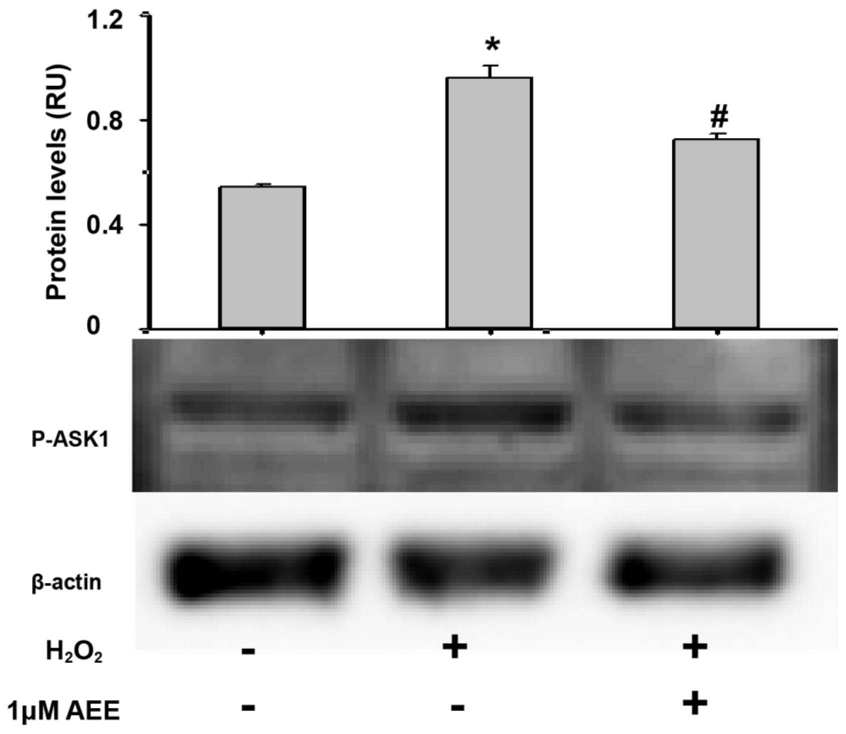

Figure S3 AEE ameliorated H_2O_2-induced changes of P-ASK1. Values are presented as the means ± SD where applicable (n = 6); all data were normalized to the corresponding control and reported in relative units (RU). *p < 0.05 compared with the normal group, #p < 0.05 compared with the H_2O_2 group. "+": with the treatments in the HUVECs; "–": without the treatments in the HUVECs.

Table S1. The level of LDH, CK, AST, ALT, BUN and Cr in rat serum

(Mean ± SD)

Group	LDH (U/L)	CK (U/L)	AST (U/L)	ALT (U/L)	BUN (mM)	Cr (μM)
**control**	306±17	201.6±31.5	25.4±3.2	94.2±7.3	5.7±1.9	37.9±2.6
**PQ**	721±25*	653.7±43.8*	72.5±9.5*	192.4±5.7*	22.1±4.7*	89.5±4.5*
**27 mg/kg AEE+PQ**	$608±43^{\#}$	602.2±24.5	$64.3±8.7^{\#}$	$168.5±4.9^{\#}$	17.4±3.2	79.7±5.9
**54 mg/kg AEE+PQ**	$506±31^{\#}$	$442.7±13.8^{\#}$	$47.5±7.6^{\#}$	$149.41±9.2^{\#}$	$13.7±2.1^{\#}$	$59.8±3.7^{\#}$
**108mg/kg AEE+PQ**	$572±52^{\#}$	$496.9±47.8^{\#}$	$54.8±12.5^{\#}$	$155.5±11.5^{\#}$	$16.5±2.0^{\#}$	$66.2±4.8^{\#}$

*p < 0.05 compared with the normal group, #p < 0.05 compared with the PQ group. LDH: Lactic dehydrogenase; CK: Creatine kinase; AST: Aspartate transaminase; ALT: Alanine aminotransferase; BUN: Blood urea nitrogen; Cr: Creatinine.
